# The Impact of Pediatric Opioid-Related Visits on U.S. Emergency Departments

**DOI:** 10.3390/children9040524

**Published:** 2022-04-07

**Authors:** Tiffany Champagne-Langabeer, Marylou Cardenas-Turanzas, Irma T. Ugalde, Christine Bakos-Block, Angela L. Stotts, Lisa Cleveland, Steven Shoptaw, James R. Langabeer

**Affiliations:** 1 Center for Health Systems Analytics, School of Biomedical Informatics, The University of Texas Health Science Center at Houston (UTHealth Houston), Houston, TX 77030, USA; maria.cardenasturanzas@uth.tmc.edu (M.C.-T.); christine.bakosblock@uth.tmc.edu (C.B.-B.); james.r.langabeer@uth.tmc.edu (J.R.L.); 2 Department of Emergency Medicine, McGovern Medical School, The University of Texas Health Science Center at Houston (UTHealth Houston), Houston, TX 77030, USA; irma.t.ugalde@uth.tmc.edu; 3 Department of Family and Community Medicine, McGovern Medical School, The University of Texas Health Science Center at Houston (UTHealth Houston), Houston, TX 77030, USA; angela.l.stotts@uth.tmc.edu; 4 UTHealth San Antonio, School of Nursing, San Antonio, TX 78229, USA; clevelandl@uthscsa.edu; 5 Center for Behavioral and Addiction Medicine, Department of Family Medicine, University of California, Los Angeles, CA 90024, USA; sshoptaw@mednet.ucla.edu

**Keywords:** emergency department, opioid use disorder, overdose, pediatrics, cost

## Abstract

Background: While there is significant research exploring adults’ use of opioids, there has been minimal focus on the opioid impact within emergency departments for the pediatric population. Methods: We examined data from the Agency for Healthcare Research, the National Emergency Department Sample (NEDS), and death data from the Centers for Disease Control and Prevention. Sociodemographic and financial variables were analyzed for encounters during 2014–2017 for patients under age 18, matching diagnoses codes for opioid-related overdose or opioid use disorder. Results: During this period, 59,658 children presented to an ED for any diagnoses involving opioids. The majority (68.5%) of visits were related to overdoses (poisoning), with a mean age of 11.3 years and a majority female (53%). There was a curvilinear relationship between age and encounters, with teens representing the majority of visits, followed by infants. The highest volume was seen in the Southern U.S., with over 58% more opioid visits than the next highest region (Midwest). Charges exceeded USD 157 million, representing 2% of total ED costs, with Medicaid responsible for 54% of the total. Conclusions: With increases in substance use among children, there is a growing need for pediatric emergency physicians to recognize, refer, and initiate treatments.

## 1. Introduction

Over the last two decades, opioid-related overdose (ORD) deaths among the pediatric population have increased by 268% (from 1999 to 2016), and in 2016 accounted for over 12% of all deaths among 15- to 24-year-olds [1,2]. Although the largest increase in overdose mortality rates occurred in adolescents aged 15 to 19 years, data from the Centers for Disease Control and Prevention (CDC) show trends increasing in youth aged 10 to 14 years as well. One in five children admit to using opioids, and rates of heroin and synthetic opioid (such as fentanyl) use are also increasing [1,3]. Since 2000, over 9000 children and adolescents died from opioid-related poisoning, nearly tripling the prior mortality prevalence; however, this age cohort may be under-represented in policy, treatment, and research, accentuating the need for additional research focused on this population [3]. Youth with opioid use disorders (OUD) are less likely to be prescribed and to use medications for OUD (MOUD) than adults, partially due to lack of physicians practicing in this area and concerns about the initiation of medications such as buprenorphine or methadone. A recent Annals of Emergency Medicine study showed that approximately 1% of all pediatric (under age 18) ED visits involving opioids were prescribed buprenorphine (an agonist treatment) within 30 days following an ED visit for an overdose [4]. Other non-ED estimates show as low as 25% of insured youth receive any form of treatment, ambulatory or emergent [5,6]. The reluctance for providers to use MOUD when treating children further emphasizes the need for continued research on this population to determine evidence-based best practices.

For adults, studies have been conducted to understand the impact and financial burden of opioids in U.S. hospitals. One study reported that the total estimated financial cost of opioid use and OUD, including healthcare, criminal justice, and work-related costs for American adults, was USD 1.02 trillion in 2017 [7]. Individuals with OUD tend to have more ED visits and hospital admissions than individuals without OUD and are more likely to have chronic medical conditions and co-occurring mental health diagnoses requiring treatment [8,9,10].

Despite the growing importance, very little research has been conducted on pediatric populations and the impact of the opioid epidemic. In this study, we refer to pediatrics as individuals less than 18 years of age (0–17) and include infants, children, and adolescents. In this study, we hypothesize that there is a positive association between age and prevalence. In addition to better understanding the frequency and prevalence of opioid-related pediatric emergency department (ED) visits, we aimed to estimate the financial burden placed on emergency departments in the United States.

## 2. Materials and Methods

### 2.1. Design

The study design was a retrospective, cross-sectional analysis. For most of our analyses, we used data from the National Emergency Department Sample (NEDS), which is the largest, national all-payer emergency department database. This annual database represents the most comprehensive data of emergency department visits across representative hospitals nationally. NEDS is managed by the Agency for Healthcare Research and Quality’s Healthcare Cost and Utilization Project (AHRQ HCUP) and represents a systematically stratified sample of discharge data from approximately 1000 contributing hospitals in the United States [11]. We created a merged dataset for four years of data, 2014–2017, using the International Classification of Diseases, Tenth Revision, Clinical Modification (ICD-10 CM). From this file, we identified opioid-related poisonings or overdoses with specific ICD codes T40.x. We included coded indicating poisoning by, adverse effect of, and underdosing of the following: opium (T40.0); heroin (T401); other opioids (T40.2); methadone (T40.3); other synthetic narcotics (T40.4); unspecified opioids (T40.6); and excluded T40.5 (cocaine), since it is not an opioid. Overdose-related deaths were examined by using underlying causes of death codes (X40-44, X60-64, X85, and Y10-14). Opioid use disorders (OUD) were identified from codes: F11.1 opioid abuse; F11.2 opioid dependence; and F11.9 opioid use.

Due to changes in the ICD system, from version 9 to 10, we used a crosswalk to identify relevant codes (available at www.icd10codesearch) to translate our initial ICD-10 CM codes into ICD-9 CM codes. The ICD-9 CM codes included were: 96500–96502, 96509, and 96529 as equivalent to codes T40.1–T40.4 and T40.6. There were several codes from ICD-9 CM that did not have an equivalent in the ICD-10 CM crosswalk; thus, adjustments were made. Codes 30550-53, 30400-03, 29285, and 29289 were identified as equivalent to the ICD-10 CM codes for OUD. The years included in our study had 30 diagnoses fields for every ED visit from 2014 to 2016 and 35 fields for the year 2017.

We assessed opioid-related codes among all diagnosis fields for the visits as well as for the single primary diagnosis. We utilized hospital charges as a proxy for the costs of delivering services [12]. Although de-identified, a data use agreement was submitted to AHRQ before analyzing the data, and this study was approved by the Committee for the Protection for Human Subjects at the University of Texas Health Science Center at Houston (approval no. HSC-SBMI-17-1021).

### 2.2. Variables and Measures

NEDS data were extracted for the following categories: demographics, discharge quarter, ED disposition, hospital region, primary payer, total charges for ED services, median household income (quartile) by patient’s residential zip code, and patient location. We included visits for patients 0 to 17 years of age. We excluded any patient that was 18 years or older at the time of visit. We coded gender as a binary nominal variable (female/male). Patient location codes were based on the urban-rural classification system developed by the National Centers for Health Statistics, including the following categories: (a) central counties of metropolitan areas with ≥1 million residents, (b) fringe counties of metropolitan areas with ≥1 million residents, (c) counties of metropolitan areas with 250,000 to 999,999 residents, (d) counties of metropolitan areas of 50,000 to 249,999 residents, (e) micropolitan counties (10,000–<50,000 residents), and (f) less than 10,000. The discharge quarter included four periods per year of study, or 16 total quarters. The ED disposition was classified into four categories: (a) routine discharge, to home health, transfer to short-term hospitals, skilled nursing facilities, intermediate care facilities, or others; (b) left against medical advice, or not admitted to this hospital; (c) admitted as an inpatient; and d) died in the ED.

We also reviewed total ED charges per case: this represents the amount the hospital requests from a payer for the services rendered to patients [13]. The NEDS database does not provide hospital-specific costs and charge data to prevent ratio evaluations, which makes translation between charges and actual costs difficult to estimate.

The primary expected payer of the charges included: Medicare, Medicaid, private insurance, self-pay, no charge, and other. The median household income is compiled per zip code of residency, and it is reported as four nominal categories corresponding to four quartiles, from lowest to highest income, respectively. Incomes were adjusted for 2017 USD. The American Hospital Association Annual Survey of Hospitals and the U.S. Census Bureau defined the locations of hospitals as Northeast, Midwest, South, and West regions.

### 2.3. Statistical Analyses

We applied NEDS weighting factors to estimate national representativeness for our analyses. We reported weighted data of frequencies, proportions, and measures of central tendency as well as 95% CI when appropriate. Univariate analyses were conducted to determine factors associated with differences in the years of this study. Weighted results were reported for visits with either diagnoses or principal diagnoses of ORD or OUD. We calculated the aggregated weighted sum (95% CI) of ED charges and the aggregated weighted mean ED charges (95% CI) by primary expected payer (e.g., private insurance, public insurance, or private pay). Then, we analyzed each billing quarter to identify relevant diagnoses (in the primary diagnosis field and subsequently any diagnosis field). The marginal values represent USD, and monetary calculations for the years 2014 to 2016 were adjusted for inflation to the year 2021 according to the Bureau of Labor Statistics consumer price index. All analyses were conducted using survey commands and using the variable “discharge weights” as the sampling weight. Stata I.C. (version 15, College Station, TX, USA) was utilized to conduct all analyses.

## 3. Results

Between 2014 and 2017, there were a total of 59,658 pediatric visits for ages 0–17. Just over 68% of these visits were for opioid-related overdoses, while approximately 32% were for other diagnoses involving opioid disorders. There were 110 million total visits, which suggests that opioid-related visits are relatively rare, albeit significant (approximately 0.1% of all visits). CDC national death data for this population indicates 1361 deaths for 0–17 years of age. Of these, only 39% occurred in a hospital facility, although NEDS data suggest a far lower number (32). Table 1 presents the frequency of patient visits by year and in total.

**Figure 1:**
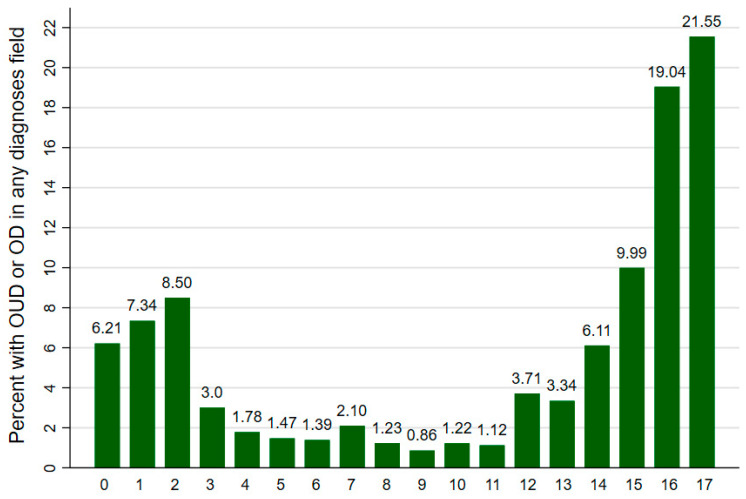
Age at visit to emergency department, all pediatric opioid-related cases, 2014–2017. Source: NEDS 2014–2017.

Approximately 53.1% of the ED visits were for female patients. The mean age of patients in this sample was 11.3 years. We observed a curvilinear relationship between age and frequency of encounters. Ages 14 to 17 had the highest overall frequency of visits (56% of total), followed by ages 0–2 (22%). Figure 1 shows the frequency by age. Table 2 summarizes the descriptive characteristics of our sample.

**Figure 2:**
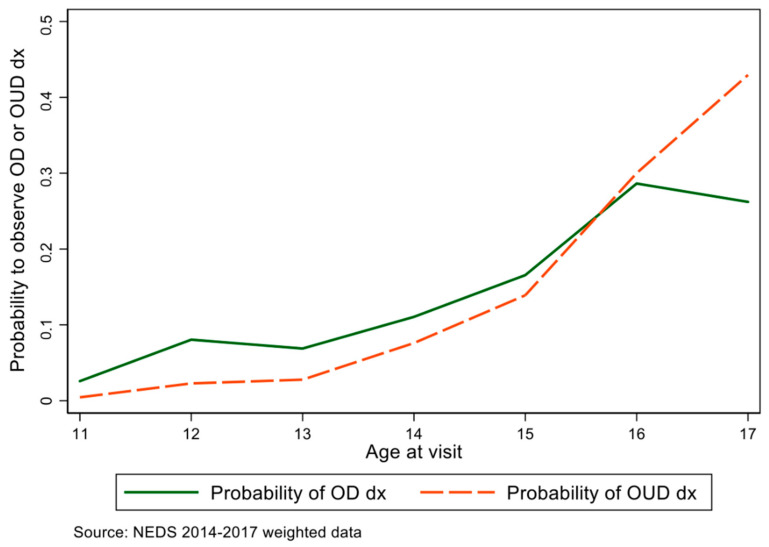
Increasing probability of an opioid-related visit, age 11–17. Source: NEDS 2014–2017, weighted data. OD, overdose; OUD, opioid use disorder; dx, diagnosis.

We also noted an increasing probability of an opioid-related visit after age 10. There was a 0.2% chance of a visit being related to opioids at age 14, which increased 50% to 0.31% at age 15, and then doubled at age 17 (67%). Age was positively associated with increased visits for both overdoses and other opioid disorders. Figure 2 summarizes the probabilities. Just over 37% of all visits occurred in the South region, the highest of the four, followed by the Midwest (with 23.6%). The Northeast had the lowest overall number of visits (16.9%). Approximately 75% of all visits occurred in large metropolitan areas with greater than 250,000 residents.

The total aggregated charges of ED visits for any diagnosis of ORD or OUD occurring from 2014 to 2017 were approximately USD 157.4 million (Table 3). Total charges across all pediatric visits for all visit types were USD 7.8 billion, indicating that charges for opioid-related visits represented 2% of the total burden on emergency departments. Charges for overdoses represented nearly 68% of the total. Medicaid was the largest payer, accounting for 54% of total billed charges, followed by private insurance payers (36.6%). Self-pay represented less than 5% of the total.

## 4. Discussion

Our analysis confirms that nearly 60,000 opioid-related pediatric visits occurred throughout U.S. emergency departments from 2014 to 2017, or roughly 15,000 cases each year. The majority of the cases that present did not result in death at the facility, but rather deaths occurred outside of the hospital. These data points are particularly troubling given the sharp increase in the probability age curve from 14–17, which will likely continue to adulthood, causing the rise in the epidemic nationwide. These visits represent an opportunity to curb the epidemic before adulthood.

An earlier study found that roughly 3132 overdoses alone occurred annually between 2006–2012 (or 22,000 in total) [14]. We found these numbers to be significantly higher (3.25×) at roughly 10,210 average per year for overdoses (and an average of 14,915 total opioid-related visits in total per year). Children appear to be at risk for overdoses and related disorders, nearly three times that of the decade prior. This increasing trend is concerning. Part of this could be the lack of relevant treatment options for this population and the need for more ambulatory access points.

The curvilinear relationship between age and frequency is also interesting. Disorders and poisonings at younger ages (0 to 2) could potentially be related to accidental exposure to parent’s medications or neonatal abstinence syndrome [15,16,17]. Prior research has shown that pregnant individuals who use substances are highly likely to pass on disorders to their newborns and infants, which could partially explain our findings [18]. The increasing likelihood of opioid-related visits from ages 14 to 17 is also concerning and suggests a need for increased screening in the ED at the teenage years for drug use. Most exposures among children and youth, whether accidental or intentional, occur in the home. This observation underscores increases in availabilities of opioids for children more recently. Children aged 3 and under are at the greatest risk of accidental exposure and overdose, while adolescents are more likely to intentionally ingest opioids [15,16,17]. The differences in age cohorts may be explained by developmental differences. Young children (under 5) in the sensorimotor and preoperational developmental stages are more likely to place found objects into their mouths, while adolescents—who are tasked with finding new experiences and engaging in risks—are likely to intentionally engage in recreational drug use [19].

The consistent increase of opioid-related overdoses and disorders in children provides opportunities to estimate the costs to the health system. One study found that opioid-related injury resulted in over 265,000 ED visits in the U.S. during the 10-year period of 2004–2013, with nearly half of those visits deemed non-medical use [20]. Because OUD is believed to be twice as prevalent in those aged 12 to 17 than in young adults, the estimates of OUD in this cohort may be drastically under-calculated [5].

Two-thirds of adults treated for OUD initiated opioid use when they were under age 25, and one-third initiated before age 17 [21]. As we see from our age probability curve, there are significant increases from 14 to 17, which likely continue in adult years. An early intervention strategy to identify, refer, and provide treatment for children and adolescents in the ED could be beneficial. This would help curb the U.S. opioid death toll for adults. The call for OUD screening and MOUD initiation in pediatric and primary care centers has grown since the American Academy of Pediatrics endorsed the use of MOUD for youth with OUD [22]. Pediatrician emergency physicians, pediatricians, and primary care providers may be reluctant to use screening and treatments [23]. Training for these providers around opioids could be a point of intervention to increase clinicians’ comfort with prescribing MOUD for children [21,24,25,26]. Youth who receive MOUD compared to behavioral therapy alone are less likely to die or discontinue treatment and are more likely to maintain abstinence [27,28,29]. The increase in synthetic opioids, such as fentanyl, is particularly concerning as many youths turn to non-medically prescribed medications for their supply. It remains that these youths are unlikely to receive timely treatment using MOUD after an overdose [30,31].

Research supporting pediatric clinician-initiated OUD screening and referrals through community-based programs has shown promising results [26,28,32]. The data on MOUD prescriptions among youth show troubling trends in prescribing patterns and gender and race disparities. Among youth with a recent non-fatal overdose, those using heroin were less likely to receive MOUD than those using other opioids, even though they were more likely to experience a subsequent overdose [30].

With nearly 60,000 ED visits involving opioid exposure among youth and adolescents, our study underscores the need for timely interventions following ED visits. Adolescents with OUD are particularly vulnerable and less likely to achieve remission than adults. This is largely because they usually return to social environments where they have little control, where their peers or parents may use substances, or they have access to unsecured prescription drugs [33]. Additionally, training and resources for parents could also be beneficial to help provide more supportive and constructive home environments [34].

There were limitations to this study. First, an alternative approach to the study design could have included a case-control method, which might have allowed for different comparisons. In addition, the dataset, while large and comprehensive, is still a sample of visits across the United States. Additional richer data might allow for a better understanding of patterns. Despite these limitations, this study adds to the scientific literature by presenting the prevalence of opioid-related visits to U.S. emergency departments.

While our findings estimate that the costs for intervention in the hospital ED for children treated for ORD and OUD are substantial, these costs in no way approximate the costs of untreated OUD in these youth. Not counted are the costs of lost wages, lost opportunity to graduate, and loss of life, nor the costs to schools, homes, outpatient clinics, and society in general. In the absence of MOUD, these children are exposed to the same factors that led to their ED and hospital visits—which are vastly different than the developmental challenges among children who do not have ORD and OUD. Disorganizing influences of continued OUD and ORD in these children and the families in which they live greatly diminish quality of life. It is accepted that hospital ED charges outweigh costs and vary considerably depending on age range, length of hospital stay, and other direct and indirect costs. The ED visits attributed to opioid toxicity in children aged 17 years and younger which occurred between 2014 and 2017 totaled approximately USD 157.4 million in corresponding charges. Greater coordination between providers in the ED, public health, ambulatory treatment, and recovery services areas is needed to promote proactive follow-up between young patients and their families.

## 5. Conclusions

With nearly 60,000 children being treated in EDs across the U.S. for opioid-related diagnoses, our findings underscore the urgent need to develop models that provide comprehensive treatment, including a medication foundation to diminish opioid-related overdose in youth, especially in the South and Midwest regions. Consequently, more coordinated strategies around training in this area are necessary for pediatric physicians, and programs to identify at-risk youth can help through early access interventions. With increases in substance use among children, there is a growing need for pediatric emergency physicians to recognize, refer, and initiate treatments. Additional strategies and resources to support both providers and parents regarding opioids and early treatment options should be explored.

## Figures and Tables

**Table 1:** Frequency of pediatric ED visits for opioid-related disorders (OUD) and opioid overdose (OD) in any diagnosis field, 2014–2017 NEDS data.

	2014	2015	2016	2017	Total (%)
Visits for Opioid-related Disorder (F11.x) *, *n*	6140	3582	4902	4196	18,820 (31.5)
Opioid Overdose (Poisoning) visits, *n*	11,026	9863	10,946	9003	40,838 (68.5)
Total OUD and ORD ED Visits, *n*	17,166	13,445	15,848	13,199	59,658 (100)

ED, emergency department; * Opioid use disorders (OUD) were identified from codes: F11.1 opioid abuse; F11.2 opioid dependence; and F11.9 opioid use.

**Table 2:** Univariate analysis of patient characteristics of weighted children visits with any relevant opioid diagnoses, 2014–2017.

Characteristic	2014, *n* (%)	2015, *n* (%)	2016, *n* (%)	2017, *n* (%)	*p*-Value *
Total (n = 59,660)	17,168 (100)	13,446 (100)	15,847 (100)	13,199 (100)	
Age, mean (sd)	11.0 (6.52)	10.62 (6.33)	11.44 (6.21)	11.33 (6.37)	
Gender					0.004
Male	8099 (47.18)	6442 (47.91)	7266 (45.85)	6154 (46.62)	
Female	9069 (52.82)	7004 (52.09)	8581 (54.15)	7045 (53.38)	
Patient location					≤0.0001
Central counties metro areas ≥1 million residents	4554 (26.60)	3469 (25.89)	4945 (31.26)	3663 (27.79)	
Fringe counties metro areas ≥1 million residents	3843 (22.45)	2907 (21.69)	3316 (20.96)	2512 (19.06)	
Counties metro areas 250,000 to 999,999 residents	4511 (26.35)	3087 (23.04)	3542 (22.39)	3336 (25.31)	
Counties metro areas 50,000 to 249,999 residents	1429 (8.35)	1372 (10.24)	1361 (8.60)	1383 (10.49)	
Micropolitan counties	1647 (9.62)	1556 (11.61)	1522 (9.62)	1336 (10.14)	
Non-metro or micropolitan counties	1135 (6.63)	1010 (7.54)	1134 (7.17)	949 (7.20)	
Discharge Status, from ED					0.03
Routine discharge from ED	14,447 (84.76)	11,471 (85.32)	10,083 (63.94)	8748 (66.76)	
Admitted inpatient	2575 (15.11)	1974 (14.68)	5634 (35.73)	4311 (32.90)	
Died in ED or inpatient	22 (0.13)	NR	53 (0.34)	45 (0.34)	
Quartile of median household income for patient zip code of residency					≤0.0001
1	4558 (26.93)	3877 (29.30)	4677 (29.92)	4028 (30.78)	
2	4822 (28.49)	3267 (24.69)	4042 (25.86)	3576 (27.32)	
3	3773 (22.29)	3225 (24.37)	3653 (23.37)	3046 (23.27)	
4	3772 (22.29)	2863 (21.64)	3261 (20.86)	2439 (18.64)	
Hospital location					≤0.0001
Northeast	3136 (18.27)	875 (21.84)	2425 (15.30)	2027 (15.35)	
Midwest	4036 (23.51)	627 (15.65)	3627 (22.89)	3584 (27.15)	
South	6396 (37.26)	1423 (35.51)	6411 (40.46)	4519 (34.24)	
West	3598 (20.96)	1082 (27.0)	3384 (21.35)	3069 (23.25)	

* Pearson’s χ^2^. AMA, against medical advice; dest, destination; ED, emergency department; NR, not reported. Note: categories in rows may not add up to the total in head column due to missing data. Percentages may not add up to 100 due to rounding.

**Table 3:** Aggregated charges billed for services by primary payer during visits to emergency department of children with diagnoses codes related to overdose and OUD, 2014–2017. Source NEDS 2014–2017 weighted data.

Charges	Opioid Poisoning (Overdose) Aggregated ED Charges for 2014–2017 Visits USD * (%)	Opioid Use Disorder Aggregated ED Charges for 2014–2017 Visits USD * (%)	Total Charges USD * (%)
Total aggregated charges	118.28 (100)	56.50 (100)	USD 174.78 (100)
Primary Payer **			
Medicare	0.59 (0.50)	0.20 (0.35)	0.80 (0.46)
Medicaid	62.17 (52.56)	28.74 (50.87)	90.90 (52.0)
Private insurance	44.49 (37.61)	21.29 (37.68)	65.80 (37.65)
Self-pay	6.89 (5.82)	4.38 (7.75)	11.27 (6.45)
No charge	0.06 (0.05)	0.03 (0.05)	0.09 (0.05)
Other	3.88 (3.28)	1.77 (3.13)	5.65 (3.23)

* Adjusted to 2021 USD million, ** charges by primary payer may not add up to aggregated ED charges due to rounding; ED, emergency department; U.S., United States. Note: years 2014 to 2017 USD values were adjusted to year 2021 using the consumer price index reported by the U.S. Bureau of Labor Statistics. Note: years 2014 to 2017 USD values were adjusted to year 2021 using the consumer price index reported by the U.S. Bureau of Labor Statistics.

## Data Availability

Data available upon reasonable request to the corresponding author.
